# Treatment of Non-idiopathic, Neglected, and Recurrent Clubfoot After Surgery Using the Ponseti Method

**DOI:** 10.7759/cureus.104016

**Published:** 2026-02-21

**Authors:** Hatim Jabri, Mohammed Mezzour, Meryem Fettah, Mohammed Tazi Charki, Hicham Abdellaoui, Karima Atarraf, Moulay Abderrahmane Afifi

**Affiliations:** 1 Department of Pediatric Orthopedics and Traumatology, Hassan II University Hospital, Fez, MAR; 2 The Human Pathology, Biomedicine, and Environment Laboratory, Faculty of Medicine, Pharmacy, and Dental Medicine of Sidi Mohamed Ben Abdellah University, Fez, MAR

**Keywords:** neglected idiopathic clubfoot, non-idiopathic clubfoot, pirani score, ponseti method, recurrent clubfoot

## Abstract

Background

The Ponseti technique is widely regarded as the most effective treatment for idiopathic clubfoot. However, the results obtained with this technique in non-idiopathic, neglected, and recurrent clubfoot remain controversial.

Objective

The objective of this study was to determine the effectiveness of the Ponseti technique in non-idiopathic clubfoot.

Materials and methods

This was a prospective study including 39 patients (60 feet) with non-idiopathic clubfoot (26 cases; 41 feet), neglected idiopathic clubfoot (six cases; eight feet), and recurrent clubfoot after posteromedial release surgery (seven cases; 11 feet). Patients were evaluated at each cast session using the Pirani score. Correction followed the cavus-adductus-varus and equinus sequence. The number of casts, initial correction rate, treatment failure rate, and recurrence rate were recorded. The average follow-up period was 18 months (range, 12-30 months).

Result

The average age at the time of the first cast was 20 months, ranging from 14 days to nine years, with a male predominance, accounting for 74.3% of cases. The malformation was located on the left side in eight cases, on the right side in 10 cases, and was bilateral in 21 cases. The causes of secondary clubfoot were dominated by spina bifida in 11 cases and arthrogryposis in eight cases. The average number of casts was seven (range, 4-14 casts): seven casts (range, 5-11 casts) in neglected clubfoot, six casts (range, 4-8 casts) in recurrent clubfoot after surgery, and seven casts (range, 5-12 casts) in non-idiopathic clubfoot. Achilles tendon tenotomy or lengthening for recurrences was performed in all patients. Anterior tibial transfer was performed in four cases of persistent residual dynamic supination. In eight cases, Achilles tendon tenotomy was performed before correction of other deformities due to stagnation of the Pirani score. Four feet presented recurrence, of which two were operated on using a posteromedial approach, and the other two were reassessed using the Ponseti method, with satisfactory results. Two feet had a recurrence of equinus, which was managed by re-tenotomy of the Achilles tendon. Two cases benefited from shortening the external arch due to persistent residual adduction.

Conclusion

Treatment of non-idiopathic, neglected, and recurrent clubfoot after surgery requires more plaster cast sessions but remains a non-invasive method with satisfactory results.

## Introduction

Clubfoot is one of the most common congenital orthopedic deformities, occurring globally at 1.18 per 1000 live births [1], with idiopathic cases accounting for 80-94.5% of all clubfoot presentations [2,3]. The Ponseti method is preferred for treating idiopathic clubfoot due to its high success rate and minimal surgical interventions. However, certain subtypes come with unique challenges that may require additional treatment adaptations.

Non-idiopathic clubfoot, commonly associated with conditions such as arthrogryposis or spina bifida, is particularly complex due to intrinsic differences in muscle function, length, and pathoanatomy, often resulting in more rigid deformities. Over time, neglected idiopathic clubfoot becomes increasingly difficult to treat as rigidity worsens with age, and the child’s increasing strength makes the casting process more challenging. Recurrent clubfoot after extensive soft tissue release is frequently complicated by scarring, diminished joint mobility, and an increased probability of necessitating additional surgical procedures.

The Ponseti method has been increasingly standardized for secondary clubfoot etiologies, such as syndromic and neurological conditions [2,3]; however, its efficacy, treatment course, and recurrence risk in these complex cases are insufficiently documented. In particular, recurrent clubfoot following extensive soft-tissue release is difficult to manage, with very limited studies addressing its treatment strategies [4]. Furthermore, differences in bracing protocols, relapse management strategies, and expertise levels across centers introduce significant variability, which makes outcome comparisons between different studies challenging and prone to bias.

This observational study, conducted by our Ponseti Team at a single tertiary care center, aims to assess treatment success, recurrence rates, and the need for adjunctive procedures. It will provide some valuable insights to refine an individualized, evidence-based approach for clubfoot cases that deviate from the typical idiopathic presentation.

## Materials and methods

This was a prospective observational study conducted from January 2021 to December 2024 at the Department of Pediatric Orthopedics and Traumatology at Hassan II University Hospital, Fez, Morocco. The study was approved by the Ethics Committee, Hospitalo-Universitaire Fes (approval number: 28/2025).

Study population

Thirty-nine patients (a total of 60 feet) with clubfoot subtypes requiring specialized management were included. Exclusion criteria were idiopathic clubfoot, patients who were lost to follow-up, and unusable reports. Patients were stratified into three groups: (i) Non-idiopathic clubfoot, defined as a secondary manifestation associated with neuromuscular, genetic, or syndromic conditions. This group comprised 26 patients with 41 feet, including cases associated with spina bifida, arthrogryposis, Larsen syndrome, cerebral palsy, osteogenesis imperfecta, and popliteal pterygium syndrome; (ii) Neglected idiopathic clubfoot, defined as untreated clubfoot until walking age (1-2 years). This group comprised six patients with eight feet; (iii) Recurrent clubfoot after extensive soft tissue release. This group included seven patients with 11 feet and was defined as clubfoot previously corrected by surgery that later relapsed.

Intervention

All patients were treated using the Ponseti method, with adaptations based on foot rigidity, age, and prior interventions. Some modifications should be noted, in particular for neglected and recurrent feet after surgery, where manipulation lasted three to five minutes, and pressure was applied to the head of the talus via the thenar eminence; the cast was often reinforced with resin. The Pirani score [5] was assessed in each cast change to monitor deformity progression by one observer.

At the end of the casting phase, Achilles tenotomy or lengthening was performed for residual equinus. Achilles tendon lengthening was performed primarily in cases of recurrence after soft tissue release due to fibrosis and in cases of popliteal pterygium syndrome due to abnormal foot anatomy. Low tenotomy incisions were avoided, as they may lead to excessive ossification and complications. Post-tenotomy casting was maintained for three to four weeks in dorsiflexion.

After cast removal, an abduction orthosis was prescribed for 23 hours/day for 3-12 months, then nighttime wear for up to five years. If abduction bracing was not tolerated, an ankle-foot orthosis was used.

Patients were followed for a minimum of 12 months; the mean follow-up was 18 months, ranging from 12 to 30 months. Outcomes were assessed based on the number of casts required for correction, the correction rates, the ability to maintain a plantigrade foot position, the recurrence rates, and the need for additional surgical procedures such as tendon transfers, osteotomies, and posterior releases. Successful correction was defined as a plantigrade, painless foot that permitted the use of standard footwear without the requirement for further surgical intervention. 

We descriptively analyzed the data, evaluating treatment response and recurrence trends across the different subtypes of clubfoot. Bracing compliance was closely monitored, given its strong correlation with relapse risk.

## Results

The average age of the patients was 20 months, ranging from 15 days to nine years. They were categorized into three groups: Non-idiopathic clubfoot, Neglected idiopathic clubfoot, and Recurrent clubfoot.

Non-idiopathic clubfoot

**Figure 1:**
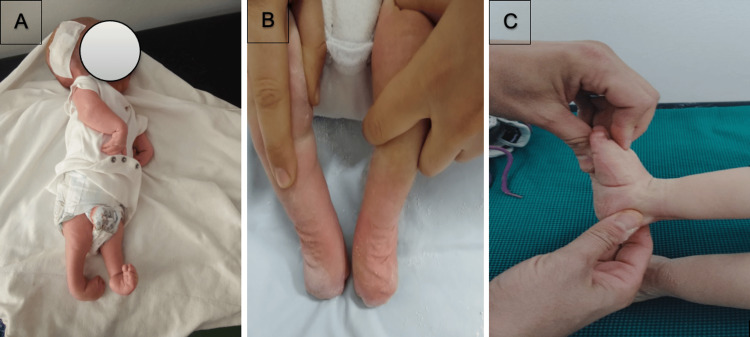
Patient with arthrogryposis and hydrocephalus (A), aspect after 10 casts (B), and aspect at the 12-month follow-up (C)

**Figure 2:**
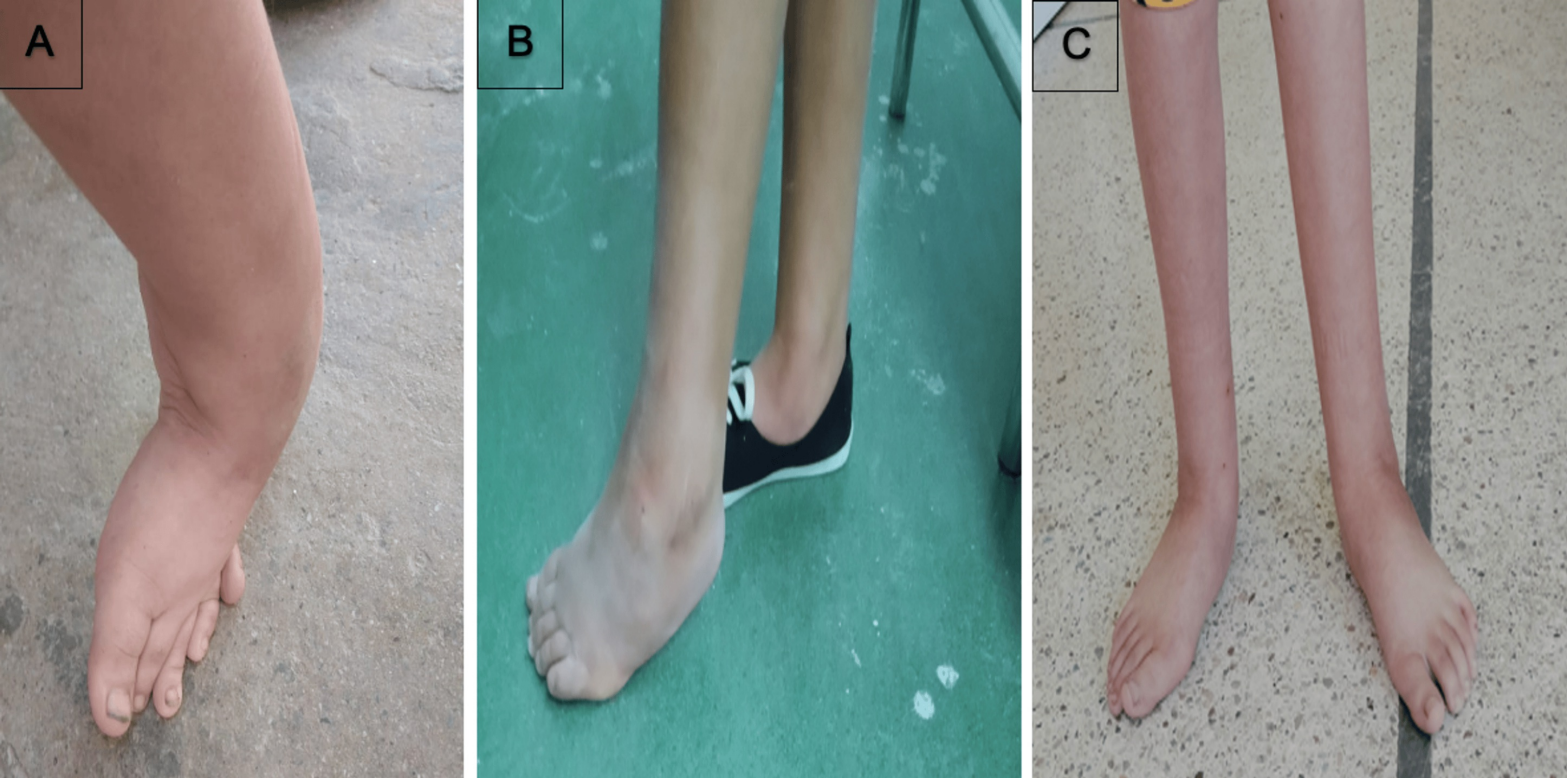
Nine-year-old girl with cerebral palsy (A), clinical aspect after five casts (B), and clinical aspect at the 20-month follow-up (C)

**Figure 3:**
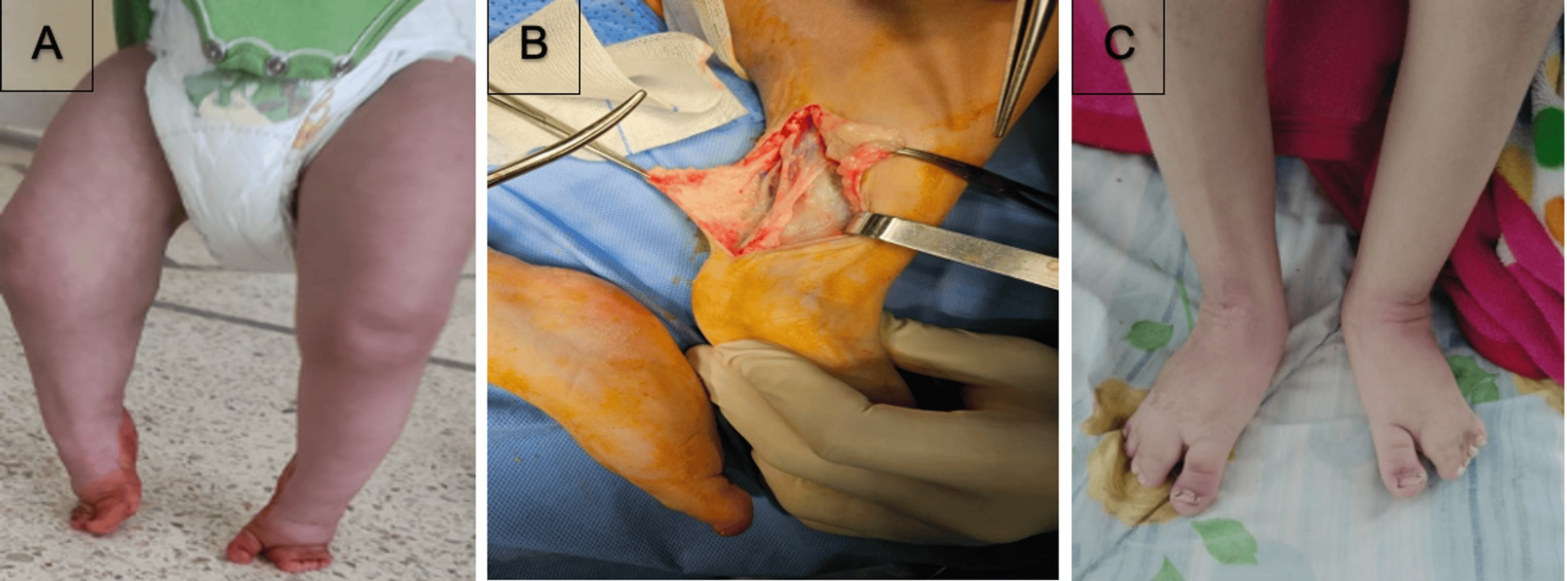
Girl with popliteal pterygium syndrome (A), achilles lengthening (B), and clinical aspect at the 18-month follow-up (C)

Of the total of 39 patients (60 feet), 26 patients (41 feet) had non-idiopathic clubfoot, including 12 patients with spina bifida and eight with arthrogryposis (Figure 1). There were also two patients with Larsen syndrome and cerebral palsy (Figure 2), while osteogenesis imperfecta and popliteal pterygium syndrome each accounted for one patient (Figure 3). The mean number of casts was seven, ranging from 4 to 14 casts. Patients with arthrogryposis required an average of 8.4 casts, ranging from 5 to 14, while patients with spina bifida needed an average of seven casts, ranging from 5 to 12. The initial correction rate, defined as the achievement of a clinically corrected foot following serial casting and Achilles tenotomy, was 86.8%, with five failures. The recurrence rate, characterized by the reappearance of at least one component of the primary deformity, was 18.2%. Early tenotomy was performed in five cases of arthrogryposis and three cases of spina bifida due to poor Pirani score improvements. Re-tenotomy was necessary for two feet, one case of arthrogryposis, and one case of spina bifida due to persistent equinus.

Neglected idiopathic clubfoot

**Figure 4:**
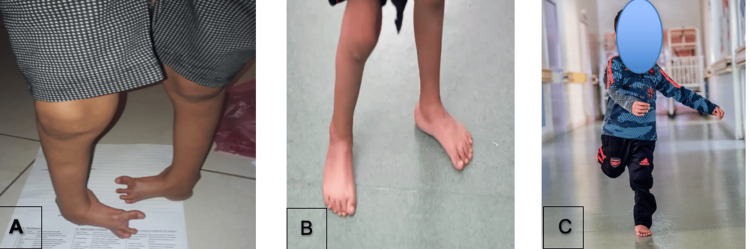
Five-year-old boy admitted for neglected clubfoot (A), appearance at two months after tenotomy (B), and appearance at the 12-month follow-up (C)

Six patients, involving eight feet, had neglected idiopathic clubfoot (Figure 4). The mean number of casts was seven, ranging from 5 to 11 casts. The initial correction rate was 100%. No recurrence was noted. All cases required Achilles tenotomy post casting, emphasizing its role in achieving full dorsiflexion. Additional procedures included tibialis anterior transfer for two feet, posterior release for two feet, and lateral column shortening for residual adduction for two feet, suggesting that residual deformities required further surgical correction.

Recurrent clubfoot

**Figure 5:**
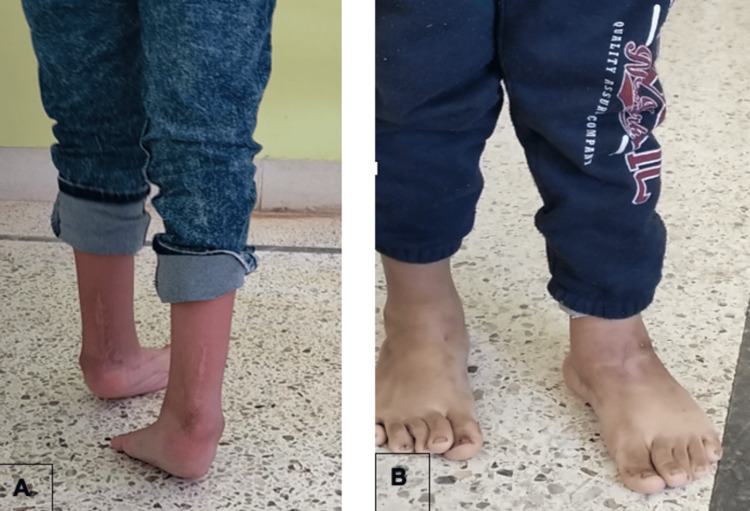
Patient with bilateral relapsed clubfoot after soft tissue release (A) and clinical aspect after five casts and lenghtening (B)

This group included seven patients with 11 feet who had recurrent clubfoot after extensive soft tissue release (Figure 5). The mean number of casts was six, ranging from four to eight casts. The initial correction rate was 85.7%, with two failures. The recurrence rate was 42.9%. Tendo-Achilles lengthening was performed due to fibrosis, reflecting the challenges of treating post-surgical stiffness. Additional procedures were performed, such as tibialis anterior transfer in two feet and lateral column shortening in one foot, suggesting a higher likelihood of requiring adjunctive procedures in this group.

The overall correction rates were calculated as follows: the initial success rate (67%) was defined as the achievement of a clinically corrected foot following serial casting and Achilles tenotomy, while the cumulative success rate (82%) included those who achieved definitive correction following secondary procedures.

## Discussion

A child's foot develops normally when the force is properly distributed through well-aligned joints, without any nerve issues or muscle problems. In untreated or inadequately managed clubfoot, ossification transpires while the bones remain misaligned and exposed to abnormal forces, which further aggravates the structural deformity of the foot.

The primary goal of congenital clubfoot treatment is to achieve a supple, functional foot that enables normal activities and shoe wear while minimizing surgical interventions. Studies have demonstrated that gait parameters, range of motion, and muscle power are superior in children treated with non-surgical or minimally invasive techniques compared to those undergoing extensive open surgical correction [6,7]. Long-term follow-up has shown that the Ponseti-treated foot functions well into middle age, with pain and mobility levels similar to those of individuals without foot deformities [8].

De Mulder et al. found in their systematic review that non-idiopathic clubfeet require more plaster cast sessions, with a success rate of 70% compared to 95% for idiopathic clubfeet [9]. Then, Abraham et al. suggest the Ponseti method as a primary approach in the initial treatment of non-idiopathic clubfoot [10].

Our findings confirm that the Ponseti method is a viable treatment for difficult-to-treat clubfoot cases that would otherwise require invasive surgical interventions. Initial correction rates were high, particularly in neglected idiopathic clubfoot (100%), followed by non-idiopathic (86.8%) and recurrent clubfoot after soft tissue release (85.7%). Nevertheless, long-term maintenance was a challenge, with an overall decrease to 68% correction at the final follow-up due to relapses, especially in the recurrent clubfoot post-soft tissue release group. These relapses were most prevalent in recurrent and non-idiopathic cases, which reflects the intrinsic rigidity and altered biomechanics of these feet.

Despite the initial success, our study highlights the sustainability issue of the Ponseti method in these very complex subtypes. The need for relapse management was frequent, often requiring more invasive interventions compared to the primary Ponseti protocol. These findings are consistent with the current literature, which indicates that recurrence rates continue to be a major issue despite the success of initial correction [11-14].

When facing a relapse, the type of recurrent deformity dictates the treatment strategy. Generally, relapses are managed with repeat manipulation and casting, following the same core principles of the initial Ponseti method [15,16]. The problem is that the most frequent relapse pattern, which is equinus, does not respond well to casting alone. In such cases, Achilles tendon lengthening or tenotomy is often required to restore a plantigrade foot. Ponseti underscored the importance of appropriate tenotomy timing, advising its execution when the anterior process of the calcaneus becomes palpable lateral to the talar head/neck [17].

Several studies have determined that bracing non-compliance is the primary factor contributing to relapse [18]. It’s essential to engage, educate, and actively involve the parents in the bracing phase to sustain correction and prevent recurrence. Each casting session should serve as an opportunity to emphasize the importance of brace compliance, the risk of relapse, and the child’s rapid foot growth over the first four to five years. Parent-focused educational interventions during the casting phase, which also help to develop an understanding of the importance of the bracing phase, have been associated with significantly lower relapse rates, reinforcing the importance of family involvement [19]. Our intervention strategies included the use of visual aids to contrast success and relapse outcomes, structured parental networking sessions, and a linguistic shift characterizing the brace as sleepwear to reduce the perceived burden on the child. 

The limitations of our study lie in its heterogeneity, with a limited number of cases, the absence of comparative statistical analysis, and a lack of long-term follow-up. 

## Conclusions

Our study reaffirms that the Ponseti method is a feasible and effective approach for treating difficult-to-manage clubfoot subtypes, including non-idiopathic, neglected, and recurrent post-surgical cases. It requires a greater number of casts but remains a minimally invasive method. However, the key to success extends beyond the initial correction and relies on long-term surveillance, early relapse detection, and parental education, which are critical to prevent recurrence and avoid more invasive procedures.
